# Assessment of Health Information Technology Interventions in Evidence-Based Medicine: A Systematic Review by Adopting a Methodological Evaluation Framework

**DOI:** 10.3390/healthcare6030109

**Published:** 2018-08-31

**Authors:** Stella C. Christopoulou, Theodore Kotsilieris, Ioannis Anagnostopoulos

**Affiliations:** 1 Department of Computer Science and Biomedical Informatics, School of Sciences, University of Thessaly, Papasiopoulou 2-4, Galaneika, Lamia 35100, Greece; janag@ucg.gr; 2 Department of Business Administration, Health & Welfare Units Administration Division, Technological Educational Institute of Peloponnese, Antikalamos, Kalamata 24100, Greece; tkots@teikal.gr

**Keywords:** Medical Subject Headings (MeSH), Health Information Technology (HIT), evidence-based medicine, Evidence-based Health Informatics (EBHI), healthcare quality, systematic review

## Abstract

Background: The application of *Health Information Technologies* (*HITs*) can be an effective way to advance medical research and health services provision. The two-fold objective of this work is to: (i) identify and review state-of-the-art *HITs* that facilitate the aims of evidence-based medicine and (ii) propose a methodology for *HIT* assessment. Methods: The systematic review was conducted according to the *Preferred Reporting Items for Systematic Reviews and Meta-Analyses* (*PRISMA*) guidelines. Furthermore, we consolidated existing knowledge in the field and proposed a *Synthesis Framework for the Assessment of Health Information Technology* (*SF/HIT*) in order to evaluate the joint use of *Randomized Controlled Trials* (*RCTs*) along with *HITs* in the field of evidence-based medicine. Results: 55 articles met the inclusion criteria and refer to 51 (*RCTs*) published between 2008 and 2016. Significant improvements in healthcare through the use of *HITs* were observed in the findings of 31 out of 51 trials—60.8%. We also confirmed that *RCTs* are valuable tools for assessing the effectiveness, acceptability, safety, privacy, appropriateness, satisfaction, performance, usefulness and adherence. Conclusions: To improve health service delivery, *RCTs* apply and exhibit formalization by providing measurable outputs. Towards this direction, we propose the *SF/HIT* as a framework which may help researchers to carry out appropriate evaluations and extend their studies.

## 1. Introduction

The application of *Health Information Technology* (*HIT*) has been proposed as a promising solution for the improvement of efficiency, effectiveness, quality of healthcare delivery. Some of the most important benefits of *HITs* are the reduction of medical errors and costs, the improvement of patients’ quality of life and the enhancement of medical decision making.

Recent research efforts have focused on the potential of *HIT* to transform the delivery of health care. Despite the fact that interoperable and multipurpose *HIT* systems have been developed, their widespread adoption in the course of care has been limited by the lack of knowledge about their types, the existence of standardized assessment frameworks/methodologies and classification methods of their outcomes that will improve health care provision. The assessment and reporting of *HIT* systems features requires standardized methods and classification procedures.

*Evidence-Based Health Informatics* (*EBHI*) can be defined as the conscientious, explicit, and judicious use of current best evidence to support a health care decision that employs *Information Technologies* (*ITs*) [1]. Towards this direction, *Randomized Controlled Trials* (*RCTs*) are considered to be a well-established experimental clinical tool, suitable not only for evaluating the efficacy of interventions, but also for supporting the conduct of an adequately designed systematic review [2].

Both *EBHI* and *RCTs* are currently at the forefront of physicians’ support for clinical decision-making. Thus, this work focuses on recent research efforts that examine whether and to what extent *HITs* are employed for evidence-based medicine purposes and, moreover, to propose a methodology for *HIT* assessment in health care delivery.

Due to the key role played by information technology in every aspect of health care, the aforementioned benefits have to be validated. Thus, we identified four broad Research Questions (RQs) that will guide the rest of our work:

- RQ1. Are there any classification methods for the detailed description of the results associated with *HITs*, and to what extent? It remains unclear whether the advances of *HITs* in recent years take into account or suggest such evaluation models or standards.
- RQ2. Is there any framework or methodology to follow for the assessment of *HITs* in order to evaluate, compare or extend results from other studies and systematic reviews? It is uncertain whether comprehensive and standardized assessment frameworks/methodologies have been proposed, or if further investigation and study is required in the field.
- RQ3. Which are the most established *HITs* (i.e., *HIT* forms in accordance with their functional capabilities and the categories of applied Information science) that support evidence-based medicine and are integrated in medical and nursing practices?
- RQ4. What are the features, the outcomes and the types of *HIT* interventions of the included studies in this research, and their classification in accordance with the medical/health domain?

## 2. Materials and Methods

The main goal of this study is to map out and propose a common and strict research method so that it leads to comparable findings among many studies. To achieve this, we used a previous systematic review of ours as a model of this study. More analytically, the two-fold objective of this article is to: (i) identify and review the state-of-the-art *HITs* with respect to the value and functionalities designed to work with them that facilitate the aims of evidence-based medicine, and (ii) propose a framework for considering the *HIT* assessment in health care delivery. As a result of these objectives, this work consists of a literature and a systematic review.

Initially, in the literature review, we examined step by step the research methodology of the articles included in our previous systematic review [3] and an unstructured preliminary evaluation framework for *HITs* emerged. Based on that work, the potential indicators (e.g., meaningfulness, relevance, usefulness, clarity, appropriateness, impact on outcome, etc.) of the proposed framework were developed and validated by a *Delphi* study [4].

More analytically, the *Delphi* method was used to collect experts’ opinions regarding key indicators (i.e., a classification list of *SF/HIT* categories/sub-categories and items) (supplementary file SF-HIT.pdf) for the *Synthesis Framework for the Assessment of Health Information Technology* (*SF/HIT*). Within this framework, two key domains (*Health domain* and *HITs*) were identified. The collection methodology of the appropriate articles was designed accordingly and the literature review (in Section 2.1) was conducted to consider related studies that employ *HIT* platforms to advance issues related with medicine and health care (addressing RQ1 and RQ2).

**Figure 1:**
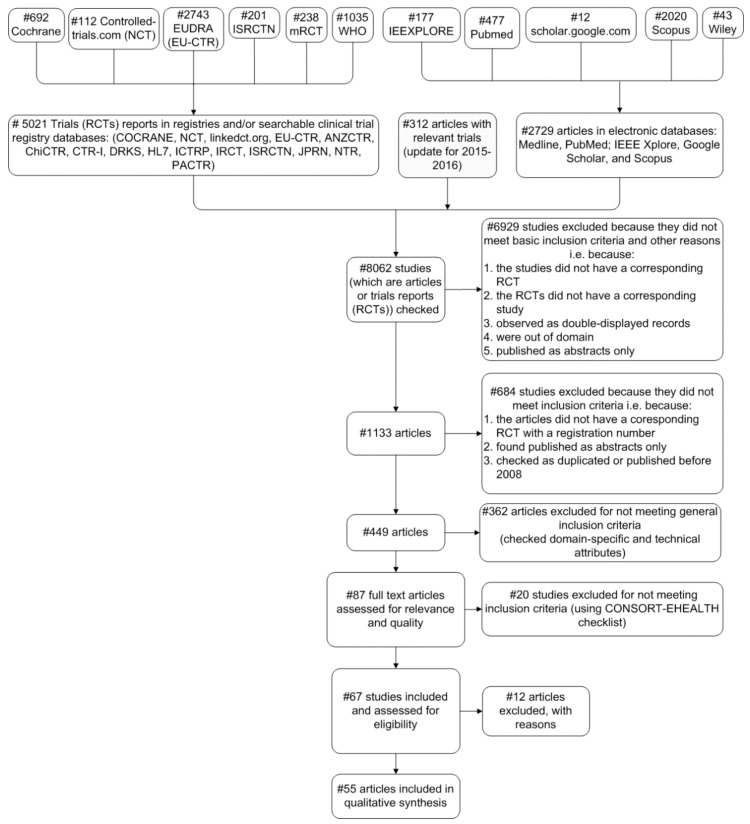
Flow chart of our study selection process (*PRISMA* 2009 flow diagram).

Thus, as depicted in Figure 1, we initially gathered the articles referred in the literature review and then we further categorized them (Tables S1 and S2 in supplementary file sup_basic.pdf) according to specific criteria as described in Section 2.2.

Then, the *SF/HIT* framework (in the form of a standard guide as presented in the supplementary file SF-HIT.pdf) was validated through a *Delphi* consultation. Subsequently, *SF/HIT* was applied in this systematic review so as to collate all evidence fitting pre-specified eligibility criteria in an attempt to address specific research questions [5].

Moreover, in order to respond to RQ3 and RQ4, we used data collected in a previous work of ours [3]. More specifically, in this systematic review, we considered only studies that involve *RCT*s, since they provide the most appropriate method for assessing the effectiveness, cost effectiveness, acceptability and safety of an intervention in evidence-based medicine [6,7,8]. The quality of our study was assessed according to the basic criteria of the *Cochrane Risk of Bias Assessment Tool* [5], while it was conducted in accordance to the *Preferred Reporting Items for Systematic Reviews and Meta-Analyses* (*PRISMA*) statement [9].

In addition, we applied the *CONSORT-EHEALTH Checklists* [10] to further assess *HITs*-based healthcare provision. Finally, we considered *RCTs* studies with at least one published article in order to compile the basic material of our study. Details about the applied methods, the electronic databases and the queries involved, as well as the data collection process are provided in our previous review [3].

### 2.1. Related Work

A short literature survey depicts *HIT* applications spanning from short message services in medical environments and high-performing healthcare systems [11,12] to tele-monitoring [13,14], e-medication treatment [15], *Electronic Health Record* (*EHR*) systems [16] and sensor technologies [17].

The evaluation of *HITs*, the so-called *Health Technology Assessment (HTA)*, is a crucial multidisciplinary activity in research and medical practice that systematically examines and evaluates the properties, effects, and/or impacts of health technology [18].

During this literature review on *HTA*, we identified several methods and taxonomies (addressing RQ1 and RQ2) that classify the assessment and properties of the evaluations of *HITs*.

The International Network of Health Technology Assessment Agencies developed a 14-item health technology assessment checklist as further support for a consistent and transparent approach of *HTA*.

Moreover, the *EUR-ASSESS* project proposed a framework for conducting and reporting *HTA* that describes a good practice in both undertaking and reporting *HTA*, while it also identifies the needs for methodologic development. This framework is complemented by specific recommendations and implementation tools, e.g., by providing the structure of the scientific summary reports and a checklist for the evaluation of methodology and the quality of *HTA* reports [19].

Also, the *EUnetHTA (European net for Healrh Technology Assessment)* project developed the *HTA* Core Model. This is a novel approach that enables effective national and transnational production and sharing of *HTA* results in a common, structured format and represents a wide range of perspectives [20].

In addition, Dixon et al., in [21], proposed a taxonomy for *HITs* on behalf of the *AHRQ’s (Agency for Healthcare Research and Quality’s) National Resource Center for HIT* in the USA. It is organized into 6 major and 28 minor categories with two additional sub-levels, forming a hierarchical and interrelated classification schema for the development, implementation, and evaluation of *HITs*. A review conducted by Jamal et al. [22] studied the use of health information technologies and systems, examining the impact of *Electronic Health Records*, *Computerized Provider Order-Entries*, and *Decision Support Systems* in medical care. Additionally, the same work examined the level of compliance in respect to evidence-based guidelines among clinicians.

Furthermore, the authors in [23] systematically evaluated the evidence on the effect of health information technology over quality, efficiency, and costs of health care. More specifically, they gathered 257 descriptive and comparative studies, as well as systematic reviews of health information technology from well-established digital libraries. As a result, the authors identified major benefits in terms of increased adherence to guideline-based care, enhanced surveillance and monitoring, and decreased medication errors. Also, the review revealed that preventive health was the main health domain in which improvement was noticed, while decreased utilization of health care services was the major benefit. Furthermore, this study acknowledges the efficacy of *HIT* for improving quality and efficiency while total development and implementation costs cannot be accurately calculated.

To accelerate the use of *HITs*, during 2009, the U.S. government introduced the *Health Information Technology for Economic and Clinical Health* (*HITECH*) Act [24]. This *Act* posed new requirements on health care organizations and professionals in terms of *meaningful-use criteria*, which drive reimbursements from the U.S. government for patient-centered care. The *meaningful-use criteria* are a set of requirements that health care organizations and professionals must meet for the adoption of *HITs*.

In the same direction, the authors of [25] examined recent evidence that correlates *HIT* functionalities (e.g., clinical decision support, computerized provider order entry, patient care reminders, e-prescribing, patient access to electronic records, etc.) with regard to quality, safety, and efficiency metrics. The authors confirm that the most important improvement incurred in *HIT* evaluations is increased measurement, analysis, and reporting of the effects of contextual and implementation factors.

The review conducted by Buntin et al. [26] included 69 articles that assessed *EHR* systems, 44 articles that studied order entry systems and 44 articles that evaluated clinical support decision systems. The authors observed that the literature was not clear in determining which criteria that strongly impact the efficient use of *HITs* had been met. However, the review revealed that the *HITs* systems improved several aspects of healthcare provision services, without noticing deterioration in any case. The study also determined that articles dealing with more than one substantial operation of *HITs* had slightly increased positive findings compared to articles that did not consider them at all. This finding is attributed to the fact that including substantial and measurable criteria makes the measurements more accurate. Consequently, the benefits of *HITs* can be determined more accurately.

Defining a common set of criteria and a strict framework (i.e., participants, type of clinical trial, medical field), the conduct of reviews and meta-analyses will provide sufficient, comparable and reliable results (over satisfaction, effectiveness, usefulness, efficiency, security, user acceptance, etc.), minimizing in parallel the risk of bias. Towards this direction, the next section proposes the framework of a classification system that enables the integration of results from multiple clinical trials and draws conclusions on the usage of *HITs*.

### 2.2. The Classification Scheme for the Synthesis of Results

In this work (addressing RQ1 and RQ3), we aim at identifying the functionalities that can be used to prototype health care delivery and to promote *health IT* evaluation.

Thus, we extend a previous work of ours [3] and we propose a solid classification schema for the development and the assessment of *HITs* in evidence-based medicine (addressing RQ2). 

The Committee on Data Standards for Patient Safety [27] classifies *EHR* systems on the basis of some core functionalities (e.g., delivery of personal health care services, care management and support processes), as well as by the supported administrative processes (e.g., billing and reimbursement). The classification criteria posed by the Committee include: patient safety, delivery of effective patient care, facilitate management of chronic conditions, efficiency and feasibility of implementation.

In another work, described in [26], *HITs* are classified by study design, care setting, health *IT* components, functions included in the meaningful-use criteria, and finally addressed outcomes. Outcomes are classified by their type (e.g., access to care, preventive care, care process, effectiveness, satisfaction, safety, etc.) and their rate (i.e., positive, mixed positive, neutral and negative).

**Figure 2:**
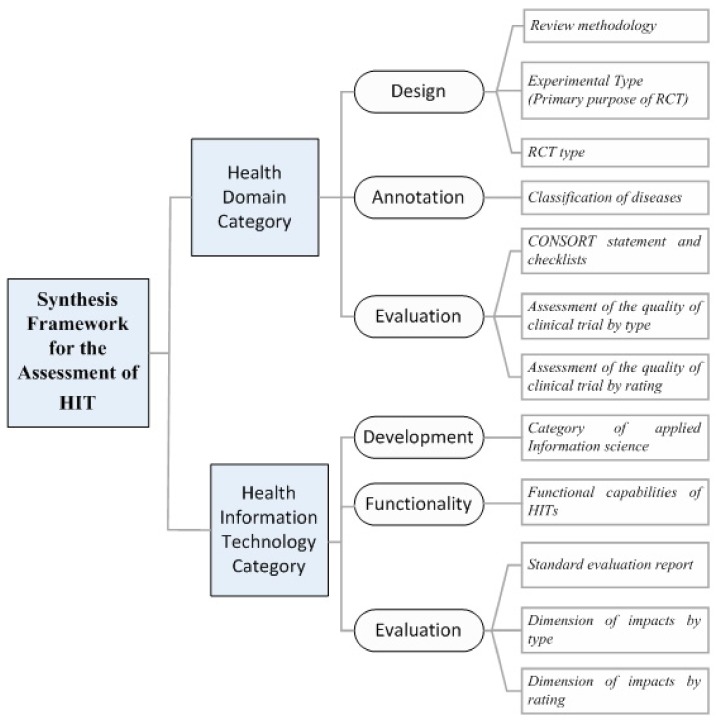
Synthesis framework for *HTA*.

With respect to RQ2, we consolidated the aforementioned existing knowledge and propose a *Synthesis Framework for the Assessment of Health Information Technology* (*SF/HIT*) (see also: supplementary file SF-HIT.pdf) for evidence-based medicine that employs *HITs* (Figure 2). *SF/HIT* consists of two main categories: (a) the *Health Core Domains category* (Table S1 in supplementary file sup_basic.pdf); and (b) the *Health Information Technology category* (Table S2 in supplementary file sup_basic.pdf). *SF/HIT* aims at filling the gap of *HITs* assessment in evidence-based medicine and towards this goal it encompasses both the technological aspect of *HITs* and the outcomes evaluation from the health domain perspective.

According to this methodology, the *Health Core Domains category* includes the findings associated with health care provision, and is divided into three sub-categories, namely (a) *design*; (b) *annotation*; and (c) *evaluation*.

In the *design* sub-category, we consider (i) the *PRISMA 2009* methodological tool [9]; (ii) the taxonomy related with the experimental type (e.g., prevention, screening, treatment, etc.) of the *RCTs* [28,29]; as well as (iii) the taxonomy related with the *RCT* type [28], e.g., open, single blind, etc.). In the *annotation* sub-category, the trials are annotated under the *ICD-10 (International Classification of Diseases—10th Revision)* medical terminology [30]. Finally, in the *evaluation* sub-category we consider (i) the *CONSORT statement and checklist* [31]; (ii) the assessment of the quality of clinical trial by type [5]; and (iii) the assessment of the quality of clinical trial by rating [5]. The first comprises a 25-item checklist and a flow diagram to provide additional guidance for *RCTs*, while the second and third are used in order to evaluate bias type (e.g., selection bias, performance bias, attrition bias, etc.) and its level (e.g., high, low, undefined) respectively.

The *Health Information Technology category* includes the findings associated with the technical aspect of the interventions. Similarly, this category contains three sub-categories, namely (a) *development*, (b) *functionality* and (c) *evaluation*.

**Figure 3:**
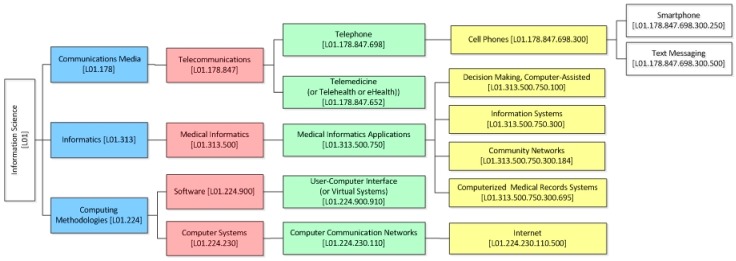
Taxonomy of *HITs* based on *MeSH* terminology.

The *development* sub-category, uses a taxonomy based on the *Medical Subject Headings* (*MeSH*) classification system [32] (Figure 3).

The functionality sub-category consists of 5 *HITs* types—CBA (Computer-Based Alerts and reminders systems), CPOE (Computerized Physician Order Entry), DSS (Decision Support Systems), EHR (Electronic Health Record), and other types—that describe the functional capabilities of *HITs*.

Finally, the *evaluation* sub-category includes (i) the *CONSORT-EHEALTH Checklists* [10]; (ii) the impacts by type; and (iii) the impacts by rating. The *CONSORT-EHEALTH Checklists* stands as a standard evaluation report, while the impacts in the reviewed studies (e.g., in preventive care, adherence/attendance, efficiency, usefulness, effectiveness, etc.) are recorded according to the suggestions described in [26].

### 2.3. Systematic Review Search Strategy

The implementation of the systematic review followed the *PRISMA 2009* flow diagram (Figure 1), and the review authors conducted the initial survey for the collection of the clinical trials and articles using the registry platforms and electronic databases.

The clinical registry platforms we used were the following: Australian and New Zealand Clinical Trials Registry, Cochrane Central Register of Controlled Trials, WHO International Clinical Trials Registry Platform, The European Clinical Trials Database, Chinese Clinical trial registry, Clinical Trials Registry—India, Deutsches Register Klinischer Studien (German Clinical Trials Register), Health Level 7, International Clinical Trials Registry Platform, Iranian Registry of Clinical Trials, International Standardised Randomised Controlled Trial Number, Japan Primary Registries Network, The Netherlands National Trial Register, Pan African Clinical Trials Registry, United States Trials Registry, European Clinical Trials Database, Linked Clinical Trials. Also, the search strategy was performed over the following academic digital libraries: Medline/PubMed, IEEE Xplore, World Health Organization library databases, Google Scholar, Wiley Online Library and Scopus.

#### 2.3.1. Eligibility Criteria

For compiling a solid basis of articles that fall within the concept of *HIT* interventions, we performed single as well as multi-term queries combined with *Boolean operators*. In order not to overlook critical synonyms in our queries, we firstly included all related *MeSH* terms (initial term set). After having collected a significant number of articles from our queried sources, we excluded keywords and terms which appeared rarely, while we added synonyms that appeared with high frequency and were not included in our initial term set. We finally ended on the following keywords and subjects: “*computer-interpretable, computer-based, measurement, assessment, evaluation, scale, rating, inventory, monitoring, tool, e-health, telemedicine, tele-health, telecare, care plan, clinical guideline*.”

Then, we applied a two-fold eligibility criterion. We considered an article in our review if (i) it referred to at least one *RCT* and (ii) employed *ICT (Information and Communications Technology)*—based methods or uses computer-based equipment.

Figure 1 illustrates the details of our search strategy. We initially considered 8062 articles and trials, out of which 6929 were excluded for not meeting criteria 1–5 of step 1. In step 2, 1133 studies were reviewed, and 684 studies were further excluded for not meeting criteria 1–3. Finally, Figure 1 depicts the general and specific eligibility criteria of steps 3, 4, and 5 that led us to the set of the 55 examined articles. Below, we summarize briefly the criteria:

- Interventional completed trials (*RCTs*) published between 2008 and 2016;
- Relative trials are assigned an official registration number, as this is a fundamental requirement from 2004 (i.e., the *International Committee of Medical Journal Editors* announced that *RCTs* will be considered for publication only if they are registered before the enrolment of the first patient [33]);
- Results of the trials are published in at least one article that belongs to well-known and established scholar databases. Therefore, every article is related to one or more trials and vice-versa;
- Publication language is English.

Due to the heterogeneity of study designs, no meta-analyses were considered. Further details on our search strategy, so as to be replicated by a third person (according to *PRISMA checklist item 8*), are provided in our preliminary work [3].

#### 2.3.2. Data Extraction and Analysis Process

In Table 1, we depict our analytical assessment in respect to seven items of the Cochrane risk of bias summary (i.e., *Sub-category 1.3.2.* and *1.3.3. of SF/HIT*). More precisely, all the elements are classified using the rationale of the proposed *SF/HIT Framework* (see also: supplementary file SF-HIT.pdf). Therefore, for every single study/trial in this review, we considered the following categories (Tables S3 and S4 in supplementary file sup_basic.pdf):

- Category of disease/health domain (e.g., neoplasm, mental, behavioral disorders, etc.) (i.e., *Sub-category 1.2.1. of SF/HIT*).
- *RCT* type (i.e., open label, single blind, double blind, not blinded and unclear) (i.e., *Sub-category 1.1.1. of SF/HIT).*
- *RCT* Experimental type (prevention, screening, treatment, supportive care and health services research) (i.e., *Sub-category 1.1.2. of SF/HIT*).
- *HITs* Functional capabilities (e.g., computer-based alerts and reminders systems, computerized provider order entry, etc.) (i.e., *Sub-category 2.2.1. of SF/HIT)*. To define values for this output, we based on the suggestions made by the authors of [23,34].
- *ICT* category—under the *MeSH* classification system (e.g., telemedicine, medical informatics applications, etc.) (i.e., *Sub-category 2.1.1. of SF/HIT)*.
- Outcomes/impacts by type (e.g., effectiveness, acceptability, usefulness, etc.) and rating (e.g., positive, negative) (i.e., *Sub-categories 2.3.2.* and *2.3.3. of SF/HIT*).

It should be noted that different or doubtful judgments were discussed and, in case of disagreement, final decisions were recorded after voting between the review authors.

## 3. Results

Table S3 in supplementary file sup_basic.pdf and Table 2 herein provide analytical and summary information about the included studies/trials (addressing RQ3), according to the *ICD-10* classification system.

Moreover, studies/trials were further examined with respect to their: (i) *RCT* type; (ii) experimental type; (iii) *HITs* functional capabilities; and (iv) *ICT* category.

In a similar way, Table S4 in sup_basic.pdf and Table 3 herein depict detailed information and synopsis about the primary outcomes and several dimensions of the impacts by both their type and their rating.

### 3.1. Descriptive Elements of the Studies and the Types of HIT in Use

As far as RQ4 is concerned, we systematically present the descriptive elements of the studies and the types of *HIT*-based interventions of the included studies according to the *ICD-10* classification system.

● **A00–B99: Certain infectious and parasitic diseases (*5 studies*/*5 trials*):** One trial describes an interactive computer kiosk that was assessed in terms of efficiency and safety, for the management of uncomplicated urinary tract infections in emergency departments [35]. Another one proposes an *antiretroviral therapy* via mobile phones in sub-Saharan Africa [36]. By using mobile phone reminder messages, the authors of [37] enhance a follow-up medical care for children who are exposed to or infected by *HIV* in Cameroon. The evaluation is performed in terms of efficiency, efficacy and success. The authors of [38] introduce a two-way mobile phone and a text-messaging system to improve adherence to antiretroviral medication, thus enhancing the communication between people infected by *HIV* and health professionals by providing effective, ethical, and efficient remote support. Additionally, in [39], the authors assessed the social support and the impact of an Internet-based *HIV* prevention program.

The above clinical trials were conducted over samples that span between 198 and 720 participants. We estimated an average time of approximately 1.8 years between the beginning of a clinical trial (registration year) and its related publication year.

● **C00–D48: Neoplasms (*4 studies*/*4 trials*):** The trials of this domain (i) study the impact of a web-based intervention that utilizes text messages to improve cancer prevention behaviors among adolescents [40], (ii) evaluate a mobile phone-based, advanced symptom management system of chemotherapy-related toxicity [41], (iii) implement a computer-based communication service [42], and (iv) assess the effectiveness of a Web-Based Colorectal Cancer Screening Patient Decision Aid [43].

All clinical trials were conducted in medium/large samples spanning from 150 up to 3000 individuals. The mean time between the kick-off of a clinical trial (i.e., the registration year) and the publication of the related results was approximately 5.3 years.

● **E00–E90: Endocrine, nutritional and metabolic diseases (*7 studies*/*7 trials*):** The authors in [44] assess an electronic alert system to improve Dyslipidemia Treatment. In [45], the authors evaluate the efficacy and safety of a continuous glucose monitor–based system. Four studies are related to mobile/internet health interventions for patients with diabetes [46,47,48,49], while an innovative study supports the use of artificial pancreas at home as a safe and beneficial option for patients with type I diabetes [50].

Among the examined trials, 5 were conducted in samples of from 36 to 200 individuals, while 2 were conducted in larger ones (from 1485 up to 88001 individuals). Between the initiation of a clinical trial (registration year) and its related publication, the average time was approximately 1.3 years.

● **F01–F99: Mental, behavioral and neurodevelopmental disorders (*18 studies*/*18 trials*):** The trials under this category are related to *ICT*-based prevention, screening and treatment in the area of mental health. Moreover, studies involve abnormalities in cognitive processes [51,52,53], depression and anxiety [54,55,56,57,58,59,60,61]. It also includes trials related with addictions, such as smoking [62,63,64,65], alcohol use [66,67] and drugs [68].

All clinical trials were conducted over samples that vary from 50 to 4800 individuals. We recorded an average time period of nearly 2.8 years between the registration of a clinical trial and its publication year.

● **I00–I99: Diseases of the circulatory system (*6 studies*/*5 trials*):** The authors of [69,70] studied mobile-based interventions for physical activity in cardiac rehabilitation, while in [71] we reviewed a physical activity intervention for women with young children. The remaining 3 studies assessed telematic services for monitoring patients suffering from uncontrolled hypertension [72,73], as well as for interventions in stroke rehabilitation [74].

All clinical trials were conducted in samples from 170 up to 400 individuals. The mean time between the kick-off of a clinical trial (i.e., the registration year) and the respective publication year was 4.3 years.

● **J00–J99: Diseases of the respiratory system (*3 studies*/*2 trials*):** The trials of this health domain studied tele-monitoring [75] and mobile-based services [75,76] for patients suffering from asthma.

Clinical trials were conducted over a sample size from 12 to 312 individuals. We recorded an average time period of nearly 4 years between the registration of a clinical trial and its publication year.

● **Z00–Z99: Factors influencing health status and contact with health services (*8 studies*/*7 trials*):** In [77], the authors studied the *ICT*-enabled prevention through a computerized virtual advisor. One trial in two other studies [78,79] assessed *CBA* systems for treatment via Internet and mobile technologies for diet, nutrition disorders and exercise. Similarly, the same assessments via Internet and mobile technologies were performed for activities of daily living [80], as well as for the health behavior domain [81]. We also reviewed studies that assessed the role of online-based education systems. Apart from increasing symptom awareness [82], the authors of the work described in [83] compared a tablet-PC-based learning approach to conventional education for improving immunosuppression. Finally, the study described in [84] examined individuals’ engagement in an ICT-based physical activity and nutrition intervention especially for men.

All clinical trials were conducted over samples varying from 10 to 504 participants. The mean time between the initiation of the clinical trial (i.e., the registration year) and its related publication was approximately 2.9 years.

● **Other subjects (older adults, reproductive health and childbirth, screening for partner violence) (*4 studies*/*3 trials*):** Four studies in this review do not explicitly fall into the previous ICD-10 categories, but still employ *HITs*. Among them, the work described in [85] refers to older adults, [86,87] refer to antenatal care and interventions for the reduction of perinatal mortality respectively, while [88] refers to screening for partner violence.

The samples of the clinical trials span from 24 to 2550 individuals. The average time period between the registration of a clinical trial and its publication year was close to 2 years.

### 3.2. Synthesis of the Characteristics and Findings

As already mentioned, we considered 55 articles published in 42 different journals that employ 51 *RCTs* in total.

The reliability of the findings is strengthened, as most of the studies included delineate a large proportion of low-risk bias assessment, mainly in the types of selection, attrition and reporting bias (Table 1).

Although *RCTs* provide strong evidence in the scopes of the examined studies, we noticed that the average time between the registration of a clinical trial until the related publication is nearly three years. This means that apart the time needed for the final publication of each article (submission, review, revision phase, etc.), there is still a significant time period required for applying *ICTs* in clinical medicine (i.e., the management of a clinical protocol and trial) (Table 2).

Now, as far as the *Health Information Technology category* (i.e., *Section 2. of SF/HIT*) is concerned (Figure 2), our analytical findings (Table 2), with respect to RQ3, are:

*Development sub-category* (i.e., *sub-category 2.1. of SF/HIT)*: 14 trials support all types of Computer Communication Networks including Internet (*MeSH* term: *L01.224.230.11*0 (Figure 3)), of which 9 offer positive results (64.3%). Also, 4 of 6 trials that support Telemedicine (*MeSH* term: *L01.178.847.652*) offer positive results (66.7%). 26 trials support the use of Smartphones and Text Messaging technologies of information science (*MeSH* terms: *L01.178.847.698.300.250* and *L01.178.847.698.300.500*), describing 50% positive outputs. 1 of 3 trials of User-Computer Interface or Virtual Systems (*MeSH* term: *L01.224.900.910*) offer positive results (33.3%). Finally, 14 trials support all types of Medical Informatics Applications (*MeSH* term: *L01.313.500.750*), of which 9 offer positive results (64.3%) (Table 2).

According to their experimental type, 47.1% of the *RCTs* fall within the treatment type, 7.8% refer to screening type while the rest of them belong to prevention (15.7%), supportive care (15.7 %), health services research (13.7%) and none in the diagnostic type (0%) (Table 2).

*Functionality sub-category* (i.e., *sub-category 2.2. of SF/HIT*): The majority of trials (27) were related to *CBA.* 14 of them (51.9%) were assessed to have positive outputs. 6 trials support *DSS* with 100% positive results. The rest of them, i.e., *CPOE*, *EHR* and *Other* types of systems, were assessed with 50%, 50% and 68.4% positive values, respectively (Table 2). These results are also fairly similar to those reported in [25], where strong evidence supports the use of clinical *DSS* and *CPOE*. 

*Evaluation sub-category* (i.e., *sub-category 2.3. of SF/HIT*): In this sub-category we examine the following three parameters:

(a) *Standard evaluation report*: Despite the fact that *CONSORT-EHEALTH* (i.e., *sub-category 2.3.1. of SF/HIT*) improves and standardizes the evaluation reports of Web-based and mobile health interventions [10], yet we found only 3 studies [59,60,61] that comply with it.

(b) *Dimension of impacts by type* (i.e., *sub-category 2.3.2. of SF/HIT*): Similar to the outcomes of [6], our research confirmed that *RCTs* remain the *gold standard* for assessing the effectiveness, preventive care, safety/privacy, appropriateness, satisfaction, service delivery/performance, usefulness and adherence/attendance. In our study, we identified the above-mentioned outcomes as well as some more (i.e., process of service delivery/performance, acceptability, cost effectiveness, and satisfaction). Furthermore, in accordance with [19], we classified them into five major categories in accordance with safety, efficacy/effectiveness, psychological/social/ethical and organizational/professional aspects of assessment.

Moreover, with respect to RQ4, we observed positive results in: (i) preventive care (6 out of 7 trials—85.7%); (ii) acceptability (6 out of 8 trials—75%); (iii) safety/privacy/security (4 out of 6 trials—66.7%); (iv) appropriateness (2 out of 3—66.7%); (v) effectiveness (26 out of 45—57.8%); (vi) satisfaction (3 out of 5—60%); (vii) service delivery/performance (3 out of 5—60%); (viii) usefulness (4 out of 7—57.1%); and (ix) adherence/attendance (5 out of 10—50%). Lower levels of positive results appeared in cost effectiveness (only 1 out of 6 trials—16.7%) and efficiency (1 out of 3 trials—33.3%) (Table 3).

(c) *Dimension of impacts by rating* (i.e., *sub-category 2.3.3. of SF/HIT*): Although in our study we classified the effects into two main groups (positive/mixed positive (the use of *HIT*s has *Positive* or *Mixed* (at least one positive and one neutral or negative) influence on the results (impacts)) and neutral/negative (the use of *HIT*s has *Neutral* or *Negative* influence on the results (impacts)), we noticed that our findings are in line with [25], where the authors reported that 56% of their examined studies presented positive results, and 21% presented mixed-positive results. That is, in our work, similarly to [25], we identified significant improvements in the quality of healthcare provision through the use of *HITs* (31 out of 51 trials—60.8%) (Table 3). The majority of them (24 trials) belong to the experimental treatment type, while all of them were assessed as having positive summary outcomes (Table 2).

## 4. Discussion

This review outlines the environment in which *HIT* and computer-based tools are applied to support evidence-based medicine.

To assist other researchers in taking the correct actions and producing tangible and reusable results, we ensured that all relevant studies employed the same types of coding and classifications (e.g., *MeSH*, functional capabilities of *HITs*, etc.) and similar metrics (e.g., in respect to adherence, efficiency, satisfaction, etc.). 

More analytically, a detailed review methodology is provided in Section 2. As far as clinical trials (i.e., *RCTs*) are concerned, the framework is already quite strict, thus several elements described in Table 2 are already included in the relevant studies (e.g., *RCT* type and experimental type). However, we believe that it would be useful in future studies to include: (i) the functional capabilities of *HITs*; (ii) the information science domains; and (iii) categorization of the impact (e.g., adherence, efficiency) under the *ICD-10* classification scheme (Table 3).

For the purposes of this study, most of these data were gathered by the authors and many of them were approximate. Also, either heterogeneous data were provided, or they were considered to be ambiguous. For these reasons, it was not possible to undertake a meta-analysis when the results and the conclusions drawn are not completely accurate and error-free.

Therefore, for the improvement of health service delivery through the successful integration of *HITs*, researchers should provide: (a) adequate and analytical assessment outputs; (b) a common framework for the evaluation of *HITs* to enhance evaluation and comparability issues; and (c) a standardized reporting mechanism on the extensive application of *HITs* in evidence-based medicine.

Towards this direction, the *SF/HIT* framework and the relative guide were presented and validated through a *Delphi* consultation and adopted as a valuable methodology. This framework, if it is taken into account both in the design of *RCTs* and systematic reviews, will help researchers to better evaluate *HITs* and expand their reviews and/or meta-analyses.

To ensure the highest possible quality of our study, we used the following methodologies.

### 4.1. Study Quality of the Literature Review

We have applied the literature review in accordance with the *Delphi* method by means of the Practical Guidance described in [89] for the application and reporting of *Delphi* procedures that are performed in order to select healthcare quality indicators and the construction of the final *SF/HIT*.

### 4.2. Study Quality of the Systematic Review

We conduct a systematic review, since this kind of review is originally used in the medical sciences to examine the effectiveness of health-care interventions and to support the practice of evidence-based medicine. Systematic reviews involve identifying, synthesizing and assessing all available evidence, quantitative and/or qualitative, in order to generate a robust, empirically derived answer to a focused research question. They are highly valuable, improve transparency and emphasize the importance of empirical evidence over preconceived knowledge [90].

In line with the above-mentioned, we follow a sufficiently clear, systematic and thorough search strategy over multiple clinical trial registries and academic digital libraries. We also adopt the *PRISMA* review methodology to ensure the transparent and complete reporting of our systematic review 

Moreover, the systematic review quality was assessed according to basic criteria of the *Cochrane Risk of Bias Assessment Tool* [5]. The articles included in the systematic review in accordance with our respective decisions were classified into seven *Cochrane risk of bias* items, with each of them being identified in one of the following three bias risk categories: high, low or uncertain (Table 1). A summary of the *CONSORT EHEALTH Checklist* [10] was applied in order to assess the quality of health information systems.

### 4.3. Limitations on the Composition of this Research Work

In our work, we faced limitations and certain types of risks of bias with respect to the heterogeneity of measures and the diverse interpretation of results in many reviewed studies. This mainly happened due to the broad aspect of addressed issues across the different health domains and technology fields under consideration.

Thus, in order to overcome these limitations, we collected supplementary information on the design, implementation and the results of the examined clinical trial (e.g., extra articles upon the study protocol used, results stored in the related registries, etc.).

Moreover, in our research strategy, we applied a two-fold eligibility criterion where the results of the trials are available in the registries and are published also in at least one article that belongs to well-known and established scholar databases and vice versa.

Furthermore, in order to assess risk of bias in the included studies we used the Cochrane Risk of Bias Assessment Tool (see also: Section 4.2 and Table 1).

Besides minimizing the selection bias of our systematic review, we used the PRISMA method (also see: Section 4.2, Flow diagram in Figure 1 and *PRISMA 2009 Checklist* in the Supplementary Materials).

Finally, as Buntin et al. [26] identified, including substantial and tangible criteria in the studies makes the measurements more accurate. Thus, following the *SF/HIT* framework, the conducting of reviews and meta-analyses will provide sufficient, comparable and reliable results, thus minimizing any type of risk of bias by defining a common set of criteria (e.g., participants, type of clinical trial, medical domain, technological field, etc.) that support measurements by type and by rate.

## 5. Conclusions

According to our findings, it appears that research surrounding Internet, telemedicine, smartphones and text messaging technologies, user-computer interfaces and virtual systems, as well as medical informatics applications, dominates the literature. Most of them are related to the development of *CBA*, *CPOE* and *EHR* systems. In addition, *RCTs* study mental, behavioral and neurodevelopmental disorders, nutritional, metabolic and circulatory diseases, as well as factors that are related to health status communication with health services.

Nevertheless, there are some state-of-the-art *HIT*-based approaches, namely wearable biosensors, artificial intelligence and machine learning systems for health monitoring and delivery that are underrepresented in this work, mainly due to their recent appearance in the field and the necessary time before the publication of the results of an *RCT*.

Finally, to improve health service delivery, *RCTs* apply and exhibit a clear formalization by providing measurable outputs that can be compared and evaluated in respect to several *HIT*-based categories and domains. Towards this direction, we propose the *SF/H*IT framework in order to classify the findings of our study in two categories and subcategories. The use *SF/HIT* provides unique insights with respect to *HITs* and health care delivery assessment in evidence-based medicine by (i) revealing the required healthcare quality indicators (i.e., the measurements) for the implementation of an *RCT* study over *HITs*, and (ii) helping the researchers to carry out the appropriate studies and evaluations and/or meta-analyses and extend their systematic reviews.

## Figures and Tables

**Table 1:**
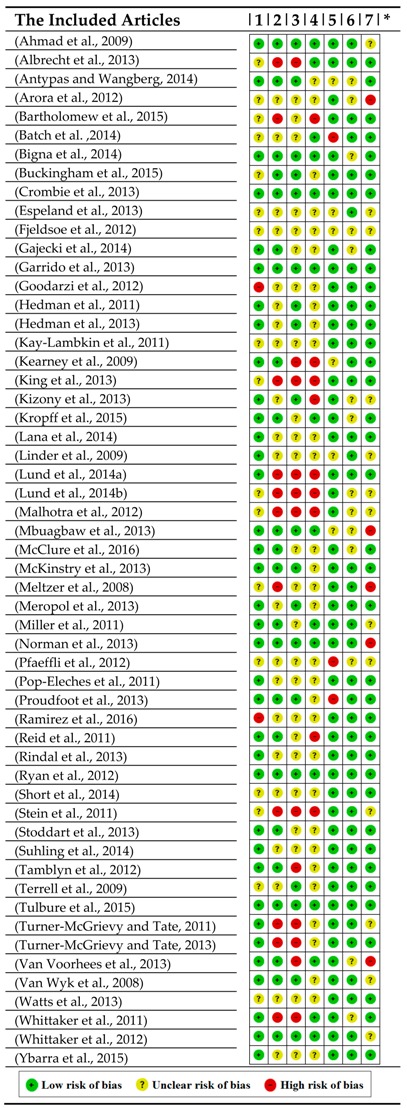
The articles included in the review and our respective decision over 7 Cochrane risk of bias items.

*: 1-Random sequence generation (selection bias); 2-Allocation concealment (selection bias); 3-Blinding of participants and personnel (performance bias); 4-Blinding of outcome assessment (detection bias); 5-Incomplete outcome data (attrition bias); 6: Selective reporting (reporting bias); 7. Other bias.

**Table 2:** Number and percentage of *RCT* type, experimental type, functional capabilities of *HITs* and categories of applied information science for the trials according to ICD-10 category.

Classification of the Studies Based on the ICD-10 Classification System	Duration (Year(s))	RCT Type					Population	Experimental Type					Functional Capabilities of HITs					Category of Applied Information Science				
		Open Label	Single Blind	Double Blind	Not Blinded	Unclear		Prevention	Screening	Treatment	Supportive Care	Health services Research	CBA	CPOE	DSS	EHR	Other	L01.224.230.110	L01.178.847.652	L01.178.847.698	L01.224.900.910	L01.313.500.750
*A00–B99: Certain infectious and parasitic diseases Infections (5 studies/5 trials)*	2.4	2	0	3	0	0	1916	1	0	2	1	1	3	0	0	0	2	1	0	3	0	1
*C00–D49: Neoplasms (4 studies/4 trials)*	5.25	0	1	0	0	3	4220	1	1	1	0	1	3	0	1	0	0	3	0	1	0	0
*E00–E89: Endocrine, nutritional and metabolic diseases (7 studies/7 trials)*	1.71	6	0	0	1	0	90,042	1	1	4	0	1	4	0	3	0	1	1	1	4	0	3
*F01-F99: Mental, Behavioral and Neurodevelopmental disorders (18 studies /18 trials)*	2.72	4	6	6	0	2	20,827	4	0	11	2	1	8	1	1	3	9	4	2	8	0	7
*I00–I99: Diseases of the circulatory system (6 studies/5 trials)*	2.60	0	1	2	0	2	1249	0	1	1	3	0	4	0	1	0	1	3	2	3	1	0
*J00–J99: Diseases of the respiratory system (3 studies/2 trials)*	3.50	1	1	0	0	0	324	0	0	1	1	0	1	0	0	0	2	0	1	1	0	0
*Z00–Z99: Factors influencing health status and contact with health services (8 studies/7 trials)*	2.86	3	1	2	0	0	1040	0	0	3	1	2	3	0	0	0	2	2	0	5	1	1
*Other subjects (Older adults, Reproductive Health and Childbirth, Screening for Partner Violence) (4 studies/3 trials)*	2.67	2	0	1	0	0	3784	0	1	1	0	1	0	1	0	0	2	0	0	1	0	2
**Number of trials**		18	11	14	1	7	-	8	4	24	8	7	27	2	6	4	19	14	6	26	3	14
**Percentage of total**		35.3%	21.6%	27.5%	2.0%	13.7%		15.7%	7.8%	47.1%	15.7%	13.7%	52.9%	3.9%	11.8%	7.8%	37.3%	27.5%	11.8%	51.0%	5.9%	27.5%
**Percentage of studies with positive outcome summary**		66.7%	63.6%	57.1 %	100 %	42.9%		75%	100%	58.3%	25%	71.4%	51.9%	50%	100%	50%	68.4%	64.3%	66.7%	50%	33.3%	64.3%
	**Summary of trials:**																			**51**		

**Table 3:** Impacts and outcomes of the trials (number and rates) according to1CD-10 category.

Classification of the Studies Based on the ICD-10 Classification System	Impact by Type											Positive Outcome Summary
	Safety	Efficacy/Effectiveness		Organizational/Professional					Psychological/Social/Ethical		Economic	
	Safety/Privacy/Security	Efficiency	Efficacy/Effectiveness	Preventive Care	Adherence/Attendance	Service Delivery/Performance	Appropriateness	Perceived Ease of Use/Usefulness	Acceptability	Satisfaction	Cost Effectiveness	
*A00–B99: Certain infectious and parasitic diseases Infections (5 studies/5 trials)*	1	2	3	1	2	1	1	0	1	1	2	1
*C00–D49: Neoplasms (4 studies/4 trials)*	0	0	3	1	1	0	0	0	0	1	0	3
*E00–E89: Endocrine, nutritional and metabolic diseases (7 studies/7 trials)*	2	0	7	1	0	2	0	2	2	1	0	7
*F01–F99: Mental, Behavioral and Neurodevelopmental disorders (18 studies/18 trials)*	0	0	16	3	4	2	0	1	4	1	1	12
*I00–I99: Diseases of the circulatory system (6 studies/5 trials)*	0	0	5	0	0	1	0	1	1	0	1	2
*J00–J99: Diseases of the respiratory system (3 studies/2 trials)*	1	0	1	0	1	0	0	1	0	0	0	0
*Z00–Z99: Factors influencing health status and contact with health services (8 studies/7 trials)*	1	1	6	1	1	0	2	2	0	1	1	4
*Other subjects (Older adults, Reproductive Health and Childbirth, Screening for Partner Violence) (4 studies/3 trials)*	1	0	2	0	1	1	0	0	0	0	0	2
**Number of trials that examined this impact**	6	3	45	7	10	7	3	7	8	5	6	
**Number wherein the examined impact contribute to positive outcome summary**	4	1	26	6	5	4	2	4	6	3	1	31
**Rate wherein the examined impact contribute to positive outcome summary**	66.7%	33.3%	57.8%	85.7%	50%	57.1%	66.7%	57.1%	75%	60%	16.7%	60.8%

## Supplementary Materials

The following are available online at http://www.mdpi.com/2227-9032/6/3/109/s1, sup_basic.pdf: supplementary file, SF-HIT.pdf: the proposed SF/HIT Framework, PRISMA.pdf: PRISMA 2009 Checklist.

## Abbreviations

**Abbreviation**	**Interpretation**
ANZCTR	Australian and New Zealand Clinical Trials Registry
AHRQ's	Agency for Healthcare Research and Quality’s
CBA	Computer-based alerts and reminders systems
ChiCTR	Chinese Clinical trial registry
ClinicalTrials.gov	United States Trials Registry
CMS	Centers for Medicare & Medicaid Services
Cochrane library	Cochrane Central Register of Controlled Trials
CONSORT	Consolidated Standards of Reporting Trials
CPOE	Computerized Physician Order Entry
CTRI	Clinical Trials Registry – India
DRKS	Deutsches Register Klinischer Studien (German Clinical Trials Register)
DSS	Decision Support Systems
EBHI	Evidence-Based Health Informatics
EHR	Electronic Health Record
EU Clinical Trials Register	European Clinical Trials Database
EudraCT	The European Clinical Trials Database
Google Scholar	Google Scholar
HIT	Health Information Technology
HITECH	Health Information Technology for Economic and Clinical Health
HL7	Health Level 7
HTA	Health Technology Assessment
ICD	International Statistical Classification of Diseases and Related Health Problems
ICMJE	International Committee of Medical Journal Editors
ICT	Information & Computer Technology
ICTRP	International Clinical Trials Registry Platform
IEEE Xplore	IEEE Xplore
IRCT	Iranian Registry of Clinical Trials
ISRCTN	International Standardised Randomised Controlled Trial Number
JPRN	Japan Primary Registries Network
LinkedCT	Linked Clinical Trials
Medline/PubMed	Medline/PubMed
MeSH	Medical Subject Headings
NTR	The Netherlands National Trial Register
PACTR	Pan African Clinical Trials Registry
PRISMA	Preferred Reporting Items for Systematic Reviews and Meta-Analyses
RCT	Randomized Controlled Trials
RQ	Research Question
Scopus	Scopus
SF/HIT	Synthesis Framework for the Assessment of Health Information Technology
WHO ICTRP	WHO International Clinical Trials Registry Platform
WHO library databases	World Health Organization library databases
Wiley Online Library	Wiley Online Library
