# Supplementary material for: Assessment of Health Information Technology Interventions in Evidence-Based Medicine: A Systematic Review by Adopting a Methodological Evaluation Framework
Source: Healthcare (Basel). 2018 Aug 31;6(3):109. doi: 10.3390/healthcare6030109 (PMC6165327; doi:10.3390/healthcare6030109)
Supplement: Supplementary file 1 [file healthcare-06-00109-s001.zip › SF-HIT.pdf]

SF/HIT Framework

SF/HIT Categories & Sub-categories			Description	Check		
1. Health Domain Category	**1.0. General**	**1.0.1.Duration**	Years/months	*Number of years/months*		
		**1.0.2, Population**[i]	Characteristics/ Number	*Individuals*		
	**1.1. Design**	**1.1.1. Comparison** (either **RCT type)**[i]	Open label			
			Single blind			
			Double blind			
			Not blinded			
			Unclear			
		**1.1.2. Experimental Type**	Prevention			
			Screening			
			Treatment			
			Supportive care			
			Health services research			
	**1.2. Annotation**	**1.2.1. ICD-10 classification (Condition**[i]**)**	A00-B99			
			……			
			Z00-Z99			
	**1.3. Evaluation**	**1.3.1. PRISMA checklist**[ii]				
		**1.3.2. CONSORT list**[iii]				
		**1.3.2. Impact by type** *(Risk of bias items* checklist)		**1.3.3. Impact by rate**		
				**High**	**Low**	**Unclear**
			Random sequence generation			
			Allocation concealment			
			Blinding of participants and personnel			
			Blinding of outcome assessment			
			Incomplete outcome data			
			Selective reporting			
			Other bias			

SF/HIT Categories & Sub-categories			Description	Check	
**2. Health Information Technology Category**	**2.1. Development**	**2.1.1. Category of applied Information science** *(Taxonomy of HITs based on MeSH terminology)*	L01.224.230.110		
			……		
			L01.313.500.750		
	**2.2. Functionality**	**2.2.1. Functional capabilities of HITs**	CBA		
			CPOE		
			DSS		
			EHR		
			Other		
	**2.3. Evaluation**	**2.3.1. CONSORT-EHEALTH list**[iii]			
		**2.3.2. Impact by type (Outcomes**[i]**)**		**2.3.3. Impact by rate**	
				**Positive or Mixed**	**Neutral or Negative**
			Preventive care		
			Adherence/Attendance		
			Efficiency		
			Perceived ease of use/Usefulness		
			Effectiveness		
			Process of service delivery/Performance		
			Safety/Privacy/Security		
			Acceptability		
			Cost effectiveness		
			Appropriateness		
			Satisfaction		

[i] In accordance with the model PICO (Patients, Intervention, Comparison, Outcomes)
[ii] Only for systematic reviews
[iii] Only for RCTs
