# Supplementary material for: Assessment of Health Information Technology Interventions in Evidence-Based Medicine: A Systematic Review by Adopting a Methodological Evaluation Framework
Source: Healthcare (Basel). 2018 Aug 31;6(3):109. doi: 10.3390/healthcare6030109 (PMC6165327; doi:10.3390/healthcare6030109)
Supplement: Supplementary file 1 [file healthcare-06-00109-s001.zip › sup_basic.pdf]

**Table S1**: Health domain categorization

| Health Domain Category | | |
|---|---|---|
| **Sub-catecory** | **Source (Reference)** | **Elements** |
| **1. DESIGN** | | |
| Review methodology | PRISMA 2009 (Moher et al., 2009) | A standard methodological tool for the design and assessment of RCTs in systematic reviews |
| Experimental Type (Primary purpose of RCT) | (National Institute of Health, 2016, 2014) | [Pr]:Prevention |
| | | [Scr]: Screening |
| | | [Tr]:Treatment |
| | | [Sup]:Supportive care |
| | | [Hsr]:Health services research |
| | | [D]:Diagnostic |
| | | [Bs]:Basic Science |
| | | [Oth]:Other |
| RCT type | (National Institute of Health, 2014) | Open |
| | | Single blind |
| | | Double blind |
| | | Unclear |
| **2. ANNOTATION** | | |
| Classification of diseases | In accordance with the medical terminology ICD-10 (WHO, 2010) | [A00-B99]:Certain infectious and parasitic diseases |
| | | [C00-D49]:Neoplasms |
| | | [E00-E89]:Endocrine, nutritional and metabolic diseases |
| | | [F01-F99]:Mental, Behavioral and Neurodevelopmental disorders |
| | | [I00-I99]:Diseases of the circulatory system |
| | | [J00-J99]:Diseases of the respiratory system, and |
| | | [Z00-Z99]:Factors influencing health status and contact with health services |
| | | Other subjects with the following sub-categories i.e., Older adults, Reproductive Health and Childbirth, Screening for Partner Violence etc. |
| **3. EVALUATION** | | |
| CONSORT statement and checklists | Trial evaluation based on (Begg et.al, 1996) | The CONSORT Statement comprises a 25-item checklist and a flow diagram. The checklist items focus on reporting how the trial was designed, analyzed, and interpreted; the flow diagram displays the progress of all participants through the trial. |
| Assessment of the quality of clinical trial by type | Bias evaluation (Higgins and Green, 2011). | Random sequence generation (selection bias) |
| | | Allocation concealment (selection bias) |
| | | Blinding of participants and personnel (performance bias) |
| | | Blinding of outcome assessment (detection bias) |
| | | Incomplete outcome data (attrition bias) |
| | | Selective reporting (reporting bias) |
| | | Other bias |
| Assessment of the quality of clinical trial by rating | Cochrane Risk of Bias Assessment Tool (Higgins and Green, 2011). | High Level |
| | | Low Level |
| | | Uncertain |

**Table S2:** Health Information Technology categorization

| Health Information Technology Category | | |
|---|---|---|
| **Sub-category** | **Source (Reference)** | **Elements (found in this study)** |
| **1. DEVELOPMENT** | | |
| Category of applied Information science | Reviewers' assessment of the applied HITs using the *MeSH* classification system (U.S. National Library of Medicine, 1999). | [L01.178.847.652]:Telemedicine (or Telehealth or eHealth)) |
| | | [L01.178.847.698]:Telephone including Cell phones (smart phones and text messaging) |
| | | [L01.313.500.750]:Medical Informatics Applications including: Decision Making, Computer-Assisted, Information systems, Community Networks, Computerized Medical Records Systems |
| | | [L01.224.230.110]:Computer Communication Networks including Internet |
| | | [L01.224.900.910]:User-Computer Interface (or Virtual Systems) |
| **2. FUNCTIONALITY** | | |
| Functional capabilities of HITs | Reviewers' assessment based on (Clancy and Clancy, 1999), (Chaudhry et al., 2006), (Centers for Medicare & Medicaid Services (CMS), 2010) and (Jones et al., 2014) | [CBA]: Computer based alerts and reminders systems |
| | | [CPOE]:Computerized Provider Order entry |
| | | [DSS]:Decision Support Systems |
| | | [EHR]:Electronic Health Record based systems |
| | | [OTHER]:Other or unable to classify technologies/systems |
| **3. EVALUATION** | | |
| Standard evaluation report | CONSORT-EHEALTH Checklists (Eysenbach and CONSORT-EHEALTH Group, 2011) | A checklist for the design and assessment of *HITs*. https://www.jmir.org/ojs/public/journals/1/CONSORT-EHEALTH-v1-6.pdf |
| Dimension of impacts by type | Reviewers' assessment about the type of outcomes based on (Buntin et al., 2011), | Preventive care |
| | | Adherence/ Attendance |
| | | Efficiency |
| | | Perceived ease of use/Usefulness |
| | | Effectiveness |
| | | Process of service delivery |
| | | Safety/Privacy/Security |
| | | Acceptability |
| | | Cost effectiveness |
| | | Appropriateness |
| | | Satisfaction |
| Dimension of impacts by rating | Reviewers' assessment about the type of outcomes based on (Buntin et al., 2011) | Positive or mixed-positive |
| | | Neutral or Not clear or Negative |

**Table S3**: Studies/trials in respect to *RCT* type, Experimental type, functional capabilities of *HITs* and Categories of applied Information science

√ This article holds the characteristic of the specific column

≫ This article refers to the same trial as the one mentioned in the article above

| Studies | Duration (Year(s)) | Registration (Year) | RCT type | | | | | Population | Experimental Type | | | | | Functional capabilities of HITs | | | | | Category of applied Information science | | | | |
|---|---|---|---|---|---|---|---|---|---|---|---|---|---|---|---|---|---|---|---|---|---|---|---|
| | | | Open Label | Single blind | Double blind | Not blinded | Unclear | | Prevention | Screening | Treatment | Supportive care | Health services research | CBA | CPOE | DSS | EHR | Other | L01.224.230.110 | L01.178.847.652 | L01.178.847.698 | L01.224.900.910 | L01.313.500.750 |
| ***A00-B99: Certain infectious and parasitic diseases Infections (5 studies /5 trials)*** | | | | | | | | | | | | | | | | | | | | | | | |
| (Stein et al., 2011) | **1** | **2010** | | | **√** | | | **400** | | | | | **√** | | | | | **√** | | | | | **√** |
| (Pop-Eleches et al., 2011) | **1** | **2010** | **√** | | | | | **720** | | | | **√** | | **√** | | | | | | | **√** | | |
| (Bigna et al., 2014) | **1** | **2013** | | | **√** | | | **224** | | | **√** | | | **√** | | | | | | | **√** | | |
| (Mbuagbaw et al., 2013) | **3** | **2010** | | | **√** | | | **198** | | | **√** | | | **√** | | | | | | | **√** | | |
| (Ybarra et al., 2015) | **6** | **2009** | **√** | | | | | **374** | **√** | | | | | | | | | **√** | **√** | | | | |
| ***Avg of duration/Total*** | **2.4** | **-** | **2** | **0** | **3** | **0** | **0** | **1916** | **1** | **0** | **2** | **1** | **1** | **3** | **0** | **0** | **0** | **2** | **1** | **0** | **3** | **0** | **1** |
| ***C00-D49: Neoplasms (4 studies /4 trials)*** | | | | | | | | | | | | | | | | | | | | | | | |
| (Miller Jr et al., 2011) | **4** | **2007** | | **√** | | | | **350** | | | | | **√** | **√** | | | | | **√** | | | | |
| (Meropol et al., 2013) | **8** | **2005** | | | | | **√** | **720** | | | **√** | | | | | **√** | | | **√** | | | | |
| (Lana et al., 2014) | **5** | **2009** | | | | | **√** | **3000** | **√** | | | | | **√** | | | | | **√** | | | | |
| (Kearney et al., 2009) | **4** | **2005** | | | | | **√** | **150** | | **√** | | | | **√** | | | | | | | **√** | | |
| ***Avg of duration/Total*** | **5.25** | **-** | **0** | **1** | **0** | **0** | **3** | **4220** | **1** | **1** | **1** | **0** | **1** | **3** | **0** | **1** | **0** | **0** | **3** | **0** | **1** | **0** | **0** |

| | | | | | | | | | | | | | | | | | | | | | | |
|---|---|---|---|---|---|---|---|---|---|---|---|---|---|---|---|---|---|---|---|---|---|---|
| ***E00-E89: Endocrine, nutritional and metabolic diseases (7 studies /7 trials)*** | | | | | | | | | | | | | | | | | | | | | | |
| (van Wyk et al., 2008) | **2** | **2006** | **√** | | | | | **88001** | | | **√** | | | **√** | | **√** | **√** | | | | | | **√** |
| (Arora et al., 2012) | **1** | **2011** | **√** | | | | | **200** | | | | | **√** | **√** | | | | | | | **√** | | |
| (Goodarzi et al., 2012) | **1** | **2011** | | | | **√** | | **100** | | | **√** | | | **√** | | | | | | | **√** | | |
| (Ramirez et al., 2016) | **3** | **2013** | **√** | | | | | **1485** | | **√** | | | | | | **√** | | | | | **√** | | |
| (Buckingham et al., 2015) | **2** | **2013** | **√** | | | | | **120** | **√** | | | | | | | **√** | | | | | | | **√** |
| (Bartholomew et al., 2015) | **2** | **2013** | **√** | | | | | **100** | | | **√** | | | | | | | **√** | **√** | **√** | **√** | | |
| (Kropff et al., 2015) | **1** | **2014** | **√** | | | | | **36** | | | **√** | | | **√** | | | | | | | | | **√** |
| ***Avg of duration/Total*** | **1.71** | **-** | **6** | **0** | **0** | **1** | **0** | **90042** | **1** | **1** | **4** | **0** | **1** | **4** | **0** | **3** | **0** | **1** | **1** | **1** | **4** | **0** | **3** |
| ***F01-F99: Mental, Behavioral and Neurodevelopmental disorders (18 studies /18 trials)*** | | | | | | | | | | | | | | | | | | | | | | |
| (Rindal et al., 2013) | **1** | **2012** | **√** | | | | | **548** | | | | | **√** | | | **√** | **√** | | | | | | **√** |
| (Espeland et al., 2013) (2 trials) | **5** | **2008** | | **√** | | | | **100** | **√** | | | | | | | | | **√** | | | | | **√** |
| | **3** | **2010** | | **√** | | | | **1600** | **√** | | | | | | | | | **√** | | | | | **√** |
| (Garrido et al., 2013) | **1** | **2012** | | **√** | | | | **135** | | | **√** | | | | | | | **√** | | | | | **√** |
| (Tamblyn et al., 2012) | **3** | **2009** | | **√** | | | | **4800** | | | **√** | | | | **√** | | **√** | | | | | | **√** |
| (Kay-Lambkin et al., 2011) | **4** | **2007** | | | **√** | | | **100** | | | **√** | | | | | | | **√** | | | | | **√** |
| (Linder et al., 2009) | **3** | **2006** | | | | | **√** | **4314** | | | **√** | | | **√** | | | **√** | | | | | | **√** |
| (Van Voorhees et al., 2013) | **4** | **2009** | | **√** | | | | **110** | | | | **√** | | | | | | **√** | **√** | | | | |
| (Hedman et al., 2011) | **2** | **2009** | **√** | | | | | **80** | | | **√** | | | | | | | **√** | **√** | | | | |
| (Hedman et al., 2013) | **4** | » | » | | | | | » | | | » | | | | | | | » | » | | | | |
| (Proudfoot et al., 2013) | **3** | **2010** | | | **√** | | | **2000** | | | | **√** | | **√** | | | | | **√** | **√** | **√** | | |
| (Whittaker et al., 2011) | **5** | **2006** | | | **√** | | | **1300** | | | **√** | | | **√** | | | | | | | **√** | | |
| (Whittaker et al., 2012) | **2** | **2009** | | | **√** | | | **790** | **√** | | | | | **√** | | | | | | | **√** | | |
| (Gajecki et al., 2014) | **0** | **2013** | | **√** | | | | **4408** | | | **√** | | | **√** | | | | | | **√** | **√** | | |
| (Reid et al., 2011) | **3** | **2008** | | | **√** | | | **200** | **√** | | | | | | | | | **√** | | | **√** | | |
| (Crombie et al., 2013) | **3** | **2010** | | | | | **√** | **50** | | | **√** | | | **√** | | | | | | | **√** | | |
| (Watts et al., 2013) | **2** | **2011** | **√** | | | | | **126** | | | **√** | | | **√** | | | | **√** | | | **√** | | |
| (Tulbure et al., 2015) | **3** | **2012** | **√** | | | | | **96** | | | **√** | | | | | | | **√** | **√** | | | | |
| (McClure et al., 2016) | **2** | **2014** | | | **√** | | | **70** | | | **√** | | | **√** | | | | | | | **√** | | |
| ***Avg of duration/Total*** | **2.72** | **-** | **4** | **6** | **6** | **0** | **2** | **20827** | **4** | **0** | **11** | **2** | **1** | **8** | **1** | **1** | **3** | **9** | **4** | **2** | **8** | **0** | **7** |

| | | | | | | | | | | | | | | | | | | | | | | | |
|---|---|---|---|---|---|---|---|---|---|---|---|---|---|---|---|---|---|---|---|---|---|---|---|
| ***I00-I99: Diseases of the circulatory system (6 studies /5 trials)*** | | | | | | | | | | | | | | | | | | | | | | | |
| (Pfaeffli et al., 2012) | **2** | **2010** | | | **√** | | | **170** | | | | **√** | | **√** | | | | | **√** | | **√** | | |
| (Antypas and Wangberg, 2014) | **4** | **2010** | | | **√** | | | **255** | | | | **√** | | **√** | | | | | **√** | | **√** | | |
| (Stoddart et al., 2013) | **5** | **2008** | | | | | **√** | **400** | | **√** | | | | **√** | | **√** | | | | **√** | | | |
| (McKinstry et al., 2013) | » | » | | | | | » | » | | » | | | | » | | » | | | | » | | | |
| (Fjeldsoe et al., 2012) | **1** | **2011** | | | | | **√** | **400** | | | | **√** | | **√** | | | | | **√** | | **√** | **√** | |
| (Kizony et al., 2013) | **1** | **2012** | | **√** | | | | **24** | | | **√** | | | | | | | **√** | | **√** | | | |
| ***Avg of duration/Total*** | **2.60** | **-** | **0** | **1** | **2** | **0** | **2** | **1249** | **0** | **1** | **1** | **3** | **0** | **4** | **0** | **1** | **0** | **1** | **3** | **2** | **3** | **1** | **0** |
| ***J00-J99: Diseases of the respiratory system (3 studies /2 trials)*** | | | | | | | | | | | | | | | | | | | | | | | |
| (Ryan et al., 2012) | **5** | **2007** | | **√** | | | | **312** | | | | **√** | | | | | | **√** | | **√** | | | |
| (Malhotra et al., 2012) | » | » | | » | | | | » | | | | » | | | | | | » | | » | | | |
| (Meltzer, Kelley and Hovell, 2008) | **2** | **2006** | **√** | | | | | **12** | | | **√** | | | **√** | | | | **√** | | | **√** | | |
| ***Avg of duration/Total*** | **3.50** | **-** | **1** | **1** | **0** | **0** | **0** | **324** | **0** | **0** | **1** | **1** | **0** | **1** | **0** | **0** | **0** | **2** | **0** | **1** | **1** | **0** | **0** |
| ***Z00-Z99: Factors influencing health status and contact with health services (8 studies /7 trials)*** | | | | | | | | | | | | | | | | | | | | | | | |
| (Suhling et al., 2014) | **3** | **2011** | **√** | | | | | **62** | | | | **√** | | | | | | **√** | | | | | **√** |
| (Turner-McGrievy and Tate, 2011) | **1** | **2010** | **√** | | | | | **104** | | | **√** | | | **√** | | | | | **√** | | **√** | **√** | |
| (Turner-McGrievy and Tate, 2013) | **3** | » | » | | | | | » | | | » | | | » | | | | | » | | » | » | |
| (Short et al., 2014) | **3** | **2011** | | | **√** | | | **504** | | | | | **√** | **√** | | | | | **√** | | **√** | | |
| (Batch et al., 2014) | **4** | **2010** | | | **√** | | | **300** | | | **√** | | | **√** | | | | | | | **√** | | |
| (Norman et al., 2013) | **6** | **2007** | **√** | | | | | **60** | | | **√** | | | | | | | | | | **√** | | |
| (Albrecht et al., 2013) | **0** | **2013** | | **√** | | | | **10** | | | | | **√** | | | | | **√** | | | **√** | | |
| (King et al., 2013) | **3** | **2010** | | **√** | | | | **40** | **√** | | | | | **√** | | | | | | | | **√** | |
| ***Avg of duration/Total*** | **2.86** | **-** | **3** | **1** | **2** | **0** | **0** | **1040** | **0** | **0** | **3** | **1** | **2** | **3** | **0** | **0** | **0** | **2** | **2** | **0** | **5** | **1** | **1** |

| *Other subjects (Older adults, Reproductive Health and Childbirth, Screening for Partner Violence) (4 studies /3 trials)* | | | | | | | | | | | | | | | | | | | | | | | |
|---|---|---|---|---|---|---|---|---|---|---|---|---|---|---|---|---|---|---|---|---|---|---|---|
| (Ahmad et al., 2009) | **4** | **2005** | √ | | | | | **280** | | √ | | | | | | | | √ | | | | | √ |
| (Terrell et al., 2009) | **3** | **2006** | | | √ | | | **954** | | | √ | | | | √ | | | | | | | | √ |
| (Lund et al., 2014a) | **1** | **2013** | √ | | | | | **2550** | | | | | √ | | | | | √ | | | √ | | |
| (Lund et al., 2014b) | ≫ | ≫ | ≫ | | | | | ≫ | | | | | ≫ | | | | | ≫ | | | | | |
| ***Avg of duration/Total*** | **2.67** | **-** | **2** | **0** | **1** | **0** | **0** | **3784** | **0** | **1** | **1** | **0** | **1** | **0** | **1** | **0** | **0** | **2** | **0** | **0** | **1** | **0** | **2** |
| | | | | | | | | | | | | | | | | | | | | | | | |
| **Number of trials** | **-** | **-** | **18** | **11** | **14** | **1** | **7** | **-** | **8** | **4** | **24** | **8** | **7** | **27** | **2** | **6** | **4** | **19** | **14** | **6** | **26** | **3** | **14** |
| **Number of trials with positive outcome summary** | | | **12** | **7** | **8** | **1** | **3** | | **6** | **4** | **14** | **2** | **5** | **14** | **1** | **6** | **2** | **13** | **9** | **4** | **13** | **1** | **9** |
| **Percentage of total** | **-** | **-** | **35.3%** | **21.6%** | **27.5%** | **2.0%** | **13.7%** | | **15.7%** | **7.8%** | **47.1%** | **15.7%** | **13.7%** | **52.9%** | **3.9%** | **11.8%** | **7.8%** | **37.3%** | **27.5%** | **11.8%** | **51.0%** | **5.9%** | **27.5%** |
| **Percentage with positive outcome summary** | | | **66.7%** | **63.6%** | **57.1 %** | **100 %** | **42.9%** | | **75%** | **100%** | **58.3%** | **25%** | **71.4%** | **51.9%** | **50%** | **100%** | **50%** | **68.4%** | **64,3%** | **66.7%** | **50%** | **33.3%** | **64.3%** |
| | | | | | | | | | | | | | | | | | | | | | **Summary of trials:** | | **51** |

**Table S4**: Primary outcomes and several dimensions of study/trial impacts by type and rating

- **√** This article holds the characteristic of the specific column
- **≫** This article refers to the same trial as the one mentioned in the article above
- **+** The use of *HIT*s has *Positive* or *Mixed* (at least one positive and one neutral or negative) influence on the results (impacts)
- **--** The use of *HIT*s has *Neutral* or *Negative* influence on the results (impacts)

| **Studies** | **Measures of the primary outcomes** | **Impact by type** | | | | | | | | | | | **Outcome summary (Total estimaton for the trial)** |
|---|---|---|---|---|---|---|---|---|---|---|---|---|---|
| | | **Preventive care** | **Adherence/Attendance** | **Efficiency** | **Perceived ease of use/Usefulness** | **Effectiveness** | **Process of service delivery/ Performance** | **Safety/Privacy/Security** | **Acceptability** | **Cost effectiveness** | **Appropriateness** | **Satisfaction** | |
| ***A00-B99: Certain infectious and parasitic diseases Infections (5 studies /5 trials)*** | | | | | | | | | | | | | |
| (Stein et al., 2011) | The proportion of subjects with treatment failure or relapse | | | | | √ | √ | √ | √ | | √ | | -- |
| (Pop-Eleches et al., 2011) | MEMS Adherence | | √ | | | | | | | | | | -- |
| (Bigna et al., 2014) | Efficacy; Efficiency | | | √ | | √ | | | | √ | | | -- |
| (Mbuagbaw et al., 2013) | Adherence | | √ | √ | | √ | | | | √ | | √ | -- |
| (Ybarra et al., 2015) | The frequency of unprotected sex | √ | | | | | | | | | | | + |
| ***Total of this category*** | | **1** | **2** | **2** | **0** | **3** | **1** | **1** | **1** | **2** | **1** | **1** | **1 +** |

| | | | | | | | | | | | | | |
|---|---|---|---|---|---|---|---|---|---|---|---|---|---|
| ***C00-D49: Neoplasms (4 studies /4 trials)*** | | | | | | | | | | | | | |
| (Miller Jr et al., 2011) | Receipt of colorectal cancer screening | | √ | | | √ | | | | | | | + |
| (Meropol et al., 2013) | Assess content of consultation by applying a coding scheme to transcripts and design yes/no questions; Satisfaction with patient-physician communication by Medical Interview Satisfaction Survey and Face Valid Survey; Assess decisional conflict by Decisional Conflict Scale; Expectations regarding potential benefits and adverse reactions associated with treatment options by Treatment Options Expectations Scale | | | | | √ | | | | | | √ | + |
| (Lana et al., 2014) | Global cancer behavioral risk indicator | √ | | | | √ | | | | | | | -- |
| (Kearney et al., 2009) | Changes in chemotherapy toxicity as a result of use of the mobile phone system and supporting information technology (IT) infrastructure | | | | | √ | | | | | | | + |
| ***Total of this category*** | | **1** | **1** | **0** | **0** | **3** | **0** | **0** | **0** | **0** | **0** | **1** | **3** + |
| ***E00-E89: Endocrine, nutritional and metabolic diseases (7 studies /7 trials)*** | | | | | | | | | | | | | |
| (van Wyk et al., 2008) | The percentage of correctly screened patients using anonymous patient record data; The percentage of correctly treated patients using anonymous patient record data. | | | | | √ | √ | | | | | | + |
| (Arora et al., 2012) | Hemoglobin A1C at 6 months | | | | | √ | √ | | | | | | + |
| (Goodarzi et al., 2012) | HgA1C;Attitude;Blood lipids; Fasting Blood Sugar; Knowledge; Practice; Self-efficacy | | | | | √ | | | | | | | + |
| (Ramirez et al., 2016) | Change from baseline in depression outcome at 6-months | | | | √ | √ | | √ | √ | | | | + |
| (Buckingham et al., 2015) | Comparison of intervention nights and control nights in which hypoglycemia occurred | √ | | | | √ | | | | | | | + |
| (Bartholomew et al., 2015) | Compliance with home blood glucose reporting | | | | √ | √ | | | | | | √ | + |
| (Kropff et al., 2015) | Percent time spent in target range (3.9-10.0 mmol/L or 70-180 mg/dL) during each study period | | | | | √ | | √ | √ | | | | + |
| ***Total of this category*** | | **1** | **0** | **0** | **2** | **7** | **2** | **2** | **2** | **0** | **0** | **1** | **7** + |
| | | | | | | | | | | | | | |

**_F01-F99: Mental, Behavioral and Neurodevelopmental disorders (18 studies /18 trials)_**

| | | | | | | | | | | | | | |
|---|---|---|---|---|---|---|---|---|---|---|---|---|---|
| (Rindal et al., 2013) | Patient report of dental providers delivery of: Screening for tobacco Discussion of specific strategies to assist the patient with quitting smoking Referral to a Quit line (toll free number for comprehensive tobacco cessation support) | | | | | √ | √ | | | | | | + |
| (Espeland et al., 2013) (2 trials) | Composite of six executive functioning and four episodic memory measures that have been previously validated.<br>Major mobility disability, defined as incapacity to walk 400 m. | √ | | | | √ | | | √ | | | | --<br>+ |
| (Garrido et al., 2013) | Change in neurocognitive outcomes | | | | | √ | | | | | | | + |
| (Tamblyn et al., 2012) | Rate of potentially inappropriate psychotropic medication | | | | | √ | | | | | | | -- |
| (Kay-Lambkin et al., 2011) | Reduction in depression scores as measured by the Beck Depression Inventory II | | √ | | | √ | | | √ | | | | + |
| (Linder et al., 2009) | The proportion of documented smokers who made contact with a smoking cessation counselor. | | | | | √ | | | | | | | -- |
| (Van Voorhees et al., 2013) | Percentage visiting the Internet site | | √ | | | | | | | | | | + |
| (Hedman et al., 2011) | Health Anxiety Inventory | | | | | √ | | | | | | | + |
| (Hedman et al., 2013) | ≫ | | | | | ≫ | | | | √ | | | ≫ |
| (Proudfoot et al., 2013) | Mean Depression Anxiety Stress Scales (DASS) score | | | | | √ | | | | | | | + |
| (Whittaker et al., 2011) | Continuous abstinence | | | | √ | √ | √ | | | | | | -- |
| (Whittaker et al., 2012) | Change in depressive symptoms as measured by the Child Depression Rating Scale-Revised (CDRS-R) | √ | | | | √ | | | √ | | | | + |
| (Gajecki et al., 2014) | Alcohol Use Disorders Identification Test (AUDIT);Change in estimated Blood Alcohol Concentration (eBAC) | | | | | √ | | | | | | | -- |
| (Reid et al., 2011) | Depressive symptoms | √ | | | | √ | | | | | | | + |
| (Crombie et al., | The change in frequency of heavy drinking | | | | | √ | | | | | | | -- |

| | | | | | | | | | | | | | |
|---|---|---|---|---|---|---|---|---|---|---|---|---|---|
| 2013) | | | | | | | | | | | | | |
| (Watts et al., 2013) | Patient Health Questionnaire-9 (PHQ-9) which is a 9 item depression scale. | | | | | √ | | | | | | | + |
| (Tulbure et al., 2015) | Leibowitz Social Anxiety Scale - Self Report (LSAS-SR); Social Phobia Inventory (SPIN) | | √ | | | √ | | | | | | √ | + |
| (McClure et al., 2016) | Point prevalence abstinence | | √ | | | √ | | | √ | | | | + |
| ***Total of this category*** | | **3** | **4** | **0** | **1** | **16** | **2** | **0** | **4** | **1** | **0** | **1** | **12 +** |
| ***I00-I99: Diseases of the circulatory system (6 studies /5 trials)*** | | | | | | | | | | | | | |
| (Pfaeffli et al., 2012) | Change in maximal oxygen uptake (VO2max) from baseline to 24 weeks. | | | | | √ | √ | | √ | | | | -- |
| (Antypas and Wangberg, 2014) | Duration and Intensity of Physical Activity | | | | √ | √ | | | | | | | -- |
| (Stoddart et al., 2013) | Average daytime systolic blood pressure at 6 months measured by ambulatory monitoring. | | | | | √ | | | | √ | | | + |
| (McKinstry et al., 2013) | ≫ | | | | | ≫ | | | | | | | ≫ |
| (Fjeldsoe et al., 2012) | Moderate to vigorous physical activity | | | | | √ | | | | | | | -- |
| (Kizony et al., 2013) | Chedoke Arm and Hand Activity Inventory (CAHAI-7) | | | | | √ | | | | | | | + |
| ***Total of this category*** | | **0** | **0** | **0** | **1** | **5** | **1** | **0** | **1** | **1** | **0** | **0** | **2 +** |
| ***J00-J99: Diseases of the respiratory system (3 studies /2 trials)*** | | | | | | | | | | | | | |
| (Ryan et al., 2012) | Change in asthma control between baseline and six months as measured by ACQ | | | | | √ | | | | √ | | | -- |
| (Malhotra et al., 2012) | ≫ | | | | | ≫ | | | | | | | ≫ |
| (Meltzer, Kelley and Hovell, 2008) | To determine subject preference of a traditional paper symptom diary vs. the VOCEL® Mobile Diary | | √ | | √ | √ | | √ | | | | | -- |
| ***Total of this category*** | | **0** | **1** | **0** | **1** | **1** | **0** | **1** | **0** | **0** | **0** | **0** | **0** |

***Z00-Z99: Factors influencing health status and contact with health services (8 studies /7 trials)***

| | | | | | | | | | | | | | |
|---|---|---|---|---|---|---|---|---|---|---|---|---|---|
| (Suhling et al., 2014) | Improvement of percentage of calcineurin inhibitor trough levels (Delta %) in target range 6 months after patient education compared to 6 months before patient education | | √ | | | √ | | | | √ | | √ | -- |
| (Turner-McGrievy and Tate, 2011) | Weight loss | | | | | √ | | | | | | | -- |
| (Turner-McGrievy and Tate, 2013) | ≫ | | | | | ≫ | | | | | | | ≫ |
| (Short et al., 2014) | Physical Activity Questionnaire; Nutrition/Food Questionnaire; Physical Activity Literacy; Questionnaire; Nutrition Literacy Questionnaire | | | | √ | √ | | | | | | | + |
| (Batch et al., 2014) | Change in body weight | | | | | √ | | | | | | | + |
| (Norman et al., 2013) | The Primary Outcome Will be the Effect of the mDIET System in Comparison to a Control Group on BMI Among Overweight Men and Women. | | | | | √ | | | | | | | -- |
| (Albrecht et al., 2013) | Learning efficiency before and after the learning phase measured with a 10-item SC-test | | | √ | | | | | | | √ | | + |
| (King et al., 2013) | Community Healthy Activities Model Program for Seniors (CHAMPS) Questionnaire; steps per day measured by accelerometer | √ | | | √ | √ | | √ | | | √ | | + |
| ***Total of this category*** | | **1** | **1** | **1** | **2** | **6** | **0** | **1** | **0** | **1** | **2** | **1** | **4 +** |

| | | | | | | | | | | | | | |
|---|---|---|---|---|---|---|---|---|---|---|---|---|---|
| ***Other subjects (Older adults, Reproductive Health and Childbirth, Screening for Partner Violence) (4 studies /3 trials)*** | | | | | | | | | | | | | |
| (Ahmad et al., 2009) | Discussion-opportunity about risk of partner abuse during physician-patient medical encounter (audiotaped data); Detection of partner abuse during physician-patient medical encounter (audio taped data) | | | | | √ | | √ | | | | | + |
| (Terrell et al., 2009) | Proportion of ED patients aged 65 and older who receive a potentially inappropriate medication | | | | | | √ | | | | | | + |
| (Lund et al., 2014a) | Skilled delivery attendance | | √ | | | √ | | | | | | | -- |
| (Lund et al., 2014b) | » | | » | | | » | | | | | | | » |
| ***Total of this category*** | | **0** | **1** | **0** | **0** | **2** | **1** | **1** | **0** | **0** | **0** | **0** | **2 +** |
| | | | | | | | | | | | | | **Total estimation** |
| **Number of trials that examined this impact** | | **7** | **10** | **3** | **7** | **45** | **7** | **6** | **8** | **6** | **3** | **5** | **31 +** |
| **Number wherein the examined impact contribute to positive outcome summary** | | **6** | **5** | **1** | **4** | **26** | **4** | **4** | **6** | **1** | **2** | **3** | |
| **Rate wherein the examined impact contribute to positive outcome summary** | | **85.7%** | **50.0%** | **33.3%** | **57.1%** | **57.8%** | **57.1%** | **66.7%** | **75.0%** | **16.7%** | **66.7%** | **60.0%** | **60.8%+** |
